# Liver-specific androgen receptor knockout attenuates early liver tumor development in zebrafish

**DOI:** 10.1038/s41598-019-46378-3

**Published:** 2019-07-23

**Authors:** Hankun Li, Yan Li, Jeng-Wei Lu, Xiaojing Huo, Zhiyuan Gong

**Affiliations:** Department of Biological Sciences, National University of Singapore, Singapore, Singapore

**Keywords:** Genetic engineering, Cancer models, Cancer genetics

## Abstract

Hepatocellular carcinoma (HCC) is one of the most severe cancer types and many genetic and environmental factors contribute to the development of HCC. Androgen receptor (AR) signaling is increasingly recognized as one of the important factors associated with HCC. Previously, we have developed an inducible HCC model in *kras* transgenic zebrafish. In the present study, to investigate the role of AR in liver tumor development, we specifically knocked out *ar* gene in the liver of zebrafish via the CRISPR/Cas9 system and the knockout zebrafish was named L-ARKO for liver-specific *ar* knockout. We observed that liver-specific knockout of *ar* attenuated liver tumor development in *kras* transgenic zebrafish at the early stage (one week of tumor induction). However, at the late stage (two weeks of tumor induction), essentially all *kras* transgenic fish continue to develop HCC irrespective of the absence or presence of *ar* gene, indicating an overwhelming role of the driver oncogene *kras* over *ar* knockout. Consistently, cell proliferation was reduced at the early stage, but not the late stage, of liver tumor induction in the *kras*/L-ARKO fish, indicating that the attenuant effect of *ar* knockout was at least in part via cell proliferation. Furthermore, androgen treatment showed acceleration of HCC progression in *kras* fish but not in *kras*/L-ARKO fish, further indicating the abolishment of *ar* signalling. Therefore, we have established a tissue-specific *ar* knockout zebrafish and it should be a valuable tool to investigate AR signalling in the liver in future.

## Introduction

Hepatocellular carcinoma (HCC) is the most common type of malignant liver cancer and causes millions of deaths annually. Several risk factors have been identified to associate with HCC, such as alcohol, hepatic toxins and hepatitis virus^1^. HCC is also a sex-biased disease as men are more prone to develop HCC than women^2^. The typical ratio of HCC between men and women is about 2:1 to 3:1^3^. In previous animal experiments, androgen has been documented to be an important factor to cause the higher incidence of HCC in males. After exposure to androgens, mice have increased incidence of liver tumor^4^. Castration or use of anti-androgen protects male rodents from liver tumor development^5^ while female rodents receiving testosterone have an increased susceptibility to hepatocarcinogenesis^6^. Moreover, clinical practice also suggests that application of androgens is associated with an increased risk of developing liver neoplasm including HCC^7,8^. For example, in male Fanconi’s anemia and aplastic anemia patients, substitute androgen intakes raised the susceptibility to develop liver neoplasm such as adenoma or HCC^9^. All these studies have suggested that androgen should be a target to study the gender disparity of HCC.

Androgens exert their effects through the activation of ARs, which are present in normal liver tissue and in HCC^10^. In addition, the expression and activation of ARs are also significantly increased in the liver tissue of rodent model during chemical-induced liver carcinogenesis^11^. AR has also been linked to HBV-induced hepatocarcinogenesis^12^. Large cohort studies have also indicated an existing synergistic effect between male gender and HBV on HCC progression^13^. Intriguingly, a study using hepatocyte-specific AR knockout mice has resulted in later and less HCC developed compared with their wildtype littermates in both HBV- and carcinogen-induced HCC^14^. In HCC cell lines, hepatic AR enhances RNA transcription of HBV via direct binding to the androgen response element on HBV genome^15^. Overexpression of functional AR in human HCC cells also resulted in the promotion of cell growth^14^. Thus, AR has played an important role in HCC, but AR-dependent sex disparity in HCC has not been well studied.

In zebrafish, there is only a single functional *ar* gene in the zebrafish genome^16^. *ar* mRNA is presented as maternal RNA from oocytes and starts to increase from 50% epiboly stage, suggesting its important role in late embryonic and early larval development^16^. In both male and female zebrafish, *ar* is widely expressed in various tissues including the brain, eyes, skin, kidney, gall bladder, gills, testis, ovary, gut, heart, muscle, spleen and liver^17,18^. Similar to the mammalian system, the most active ligand for zebrafish AR is also 11-ketotestosterone (KT11), which has similar potency to testosterone as an androgen^19^. To investigate the role of *ar* in HCC development, in the present study, we successfully developed a liver-specific *ar* knockout zebrafish by liver-specific expression of Cas9 nuclease using the CRISPR system^20^. By combination with our previously established zebrafish HCC model which has inducible expression of oncogenic *kras*^21^, we found that *ar* may help promote HCC progression at an early stage, but not at a late stage, in our *kras*-induced HCC model in zebrafish.

## Result

### Generation and characterization of liver-specific *ar* knockout (L-ARKO) transgenic zebrafish

In our previously published hepatocyte transcriptomic data derived from WT and *kras* transgenic zebrafish^22^, we found that *ar* transcripts were presented at a moderately abundant levels (1–7 transcripts per million transcript) in all samples including 6 dpf larvae and male and female adult fish (Supplementary Fig. S1A). To confirm the *ar* expression in these hepatocytes, RT-PCR was carried out and indeed that *ar* RNAs were detected in all of these hepatocyte RNA samples (Supplementary Fig. S1B).

**Figure 1:**
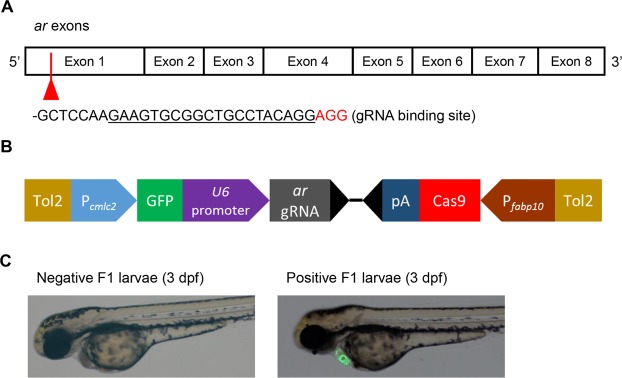
Structure of zebrafish *ar* gene and DNA construct for liver-specific knockout. (**A**) Schematic representation of eight *ar* exons to cover the complete coding region. The sequence (underlined) and position of the *ar* gRNA target at the first exon are indicated. PAM sequence (Cas9 binding site) is in Red. (**B**) Schematic illustration of the liver-specific CRISPR construct. Tol2, Tol2 transposon; P_*cmlc2*_: *cmlc2* promoter; *ar* gRNA, ar guide RNA; pA: poly A signal; P_*fabp10*_: *fabp10a* promoter. (**C**) The negative (left) and positive (right) F1 larvae from an L-ARKO founder transgenic zebrafish. The heart is marked with GFP expression as a screening marker.

With the *ar* expression in zebrafish hepatocytes ascertained, liver (hepatocyte)-specific knockout of *ar* in zebrafish was designed as illustrated in Fig. 1A,B. The DNA construct shown in Fig. 1B was injected into WT zebrafish embryos and F0 transgenic fish were screened for GFP expression in the heart at 3–5 dpf. 21.4% of F0 (112 out of 523) fish showed GFP positive phenotype (Fig. 1C). To obtain stable transgenic line with germline transmission, GFP + F0 fish were crossed with WT zebrafish and their offspring were screened for GFP expression in the heart. 7 out of 50 GFP + F0 fish had germline transmission. F1 larvae with GFP expression were collected as positive F1 fish and raised to adult (Fig. 1C). One of the F1 L-ARKO zebrafish was used to generate F2 L-ARKO zebrafish for genomic characterization. This F1 L-ARKO zebrafish was also crossed with *kras* zebrafish for the liver tumor study. Now the ARKO zebrafish have been maintained for five generations and there was no observable difference compared to wildtype zebrafish in development, growth, morphology and reproduction.

**Figure 2:**
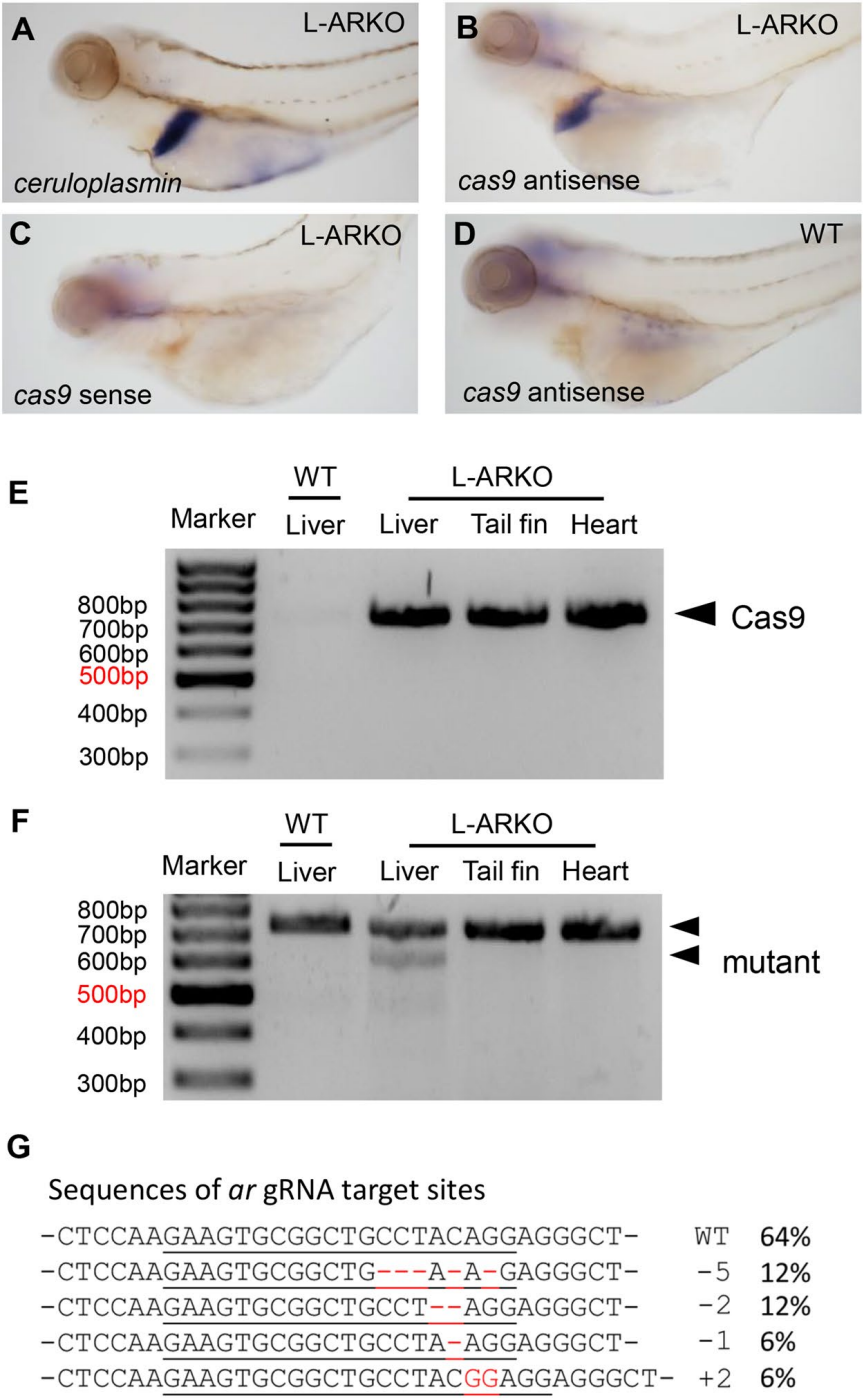
Characterization of L-ARKO zebrafish. (**A**–**D**) Images of zebrafish larvae after whole mount *in situ* hybridization. 4 dpf L-ARKO larvae were subjected to whole mount *in situ* hybridization against the *ceruloplasmin* probe (**A**), *cas9* antisense probe (**B**) and *cas9* sense probe (**C**). 4 dpf WT larvae was examined against *cas9* antisense probe (**D**). (**E**) PCR examination of *cas9* DNA in the liver, tail fin and heart of the F2 L-ARKO zebrafish. WT liver was used as a negative control. (**F**) T7E1 mutagenesis assay for the CRISPR target site in the *ar* gene in the WT zebrafish liver and F2 L-ARKO zebrafish liver, tail fin and heart. both WT and mutant *ar* bands are indicated with arrowheads. (**G**) Sequences of *ar* gRNA target sites. The target sites were amplified by PCR from liver DNA of an F2 L-ARKO fish and sequenced. The gRNA targeted sequence is underlined and the mutations in red. The type of mutation and frequency in percentage are indicated. All of the mutated sequences caused reading frame changes and premature termination codons.

To validate the *cas9* expression in the liver, *in situ* hybridization was performed on 4-dpf F1 L-ARKO fish. The probe for *ceruloplasmin*^23^ was used as a positive control. As shown in Fig. 2A, *ceruloplasmin* mRNA was specifically expressed in the liver. Similarly *cas9* expression, as assessed by a *cas9* antisense probe, was also specifically expressed in the liver region (Fig. 2B). In comparison, no hybridization signal was detected either in the L-ARKO larvae by a *cas9* sense probe (Fig. 2C) or in WT larvae by the *cas9* antisense probe (Fig. 2D).

To further characterize *ar* knockout in L-ARKO zebrafish, F2 adult L-ARKO fish were raised. First, the *cas9* DNA was detected by PCR in all tissues examined (Fig. 2E). Secondly, T7E1 mutagenesis assay of the liver from F2 L-ARKO fish showed that the mutated DNA band was present only in the liver sample (Fig. 2F). Finally, DNA sequencing of the gRNA targeted region indicated that around 36% of the alleles mutated and in total four types of mutation were identified and all of them caused reading frame shift (Fig. 2G) and introduced premature termination codons (data not shown). Thus, liver-specific knockout of *ar* was achieved in L-ARKO zebrafish.

**Figure 3:**
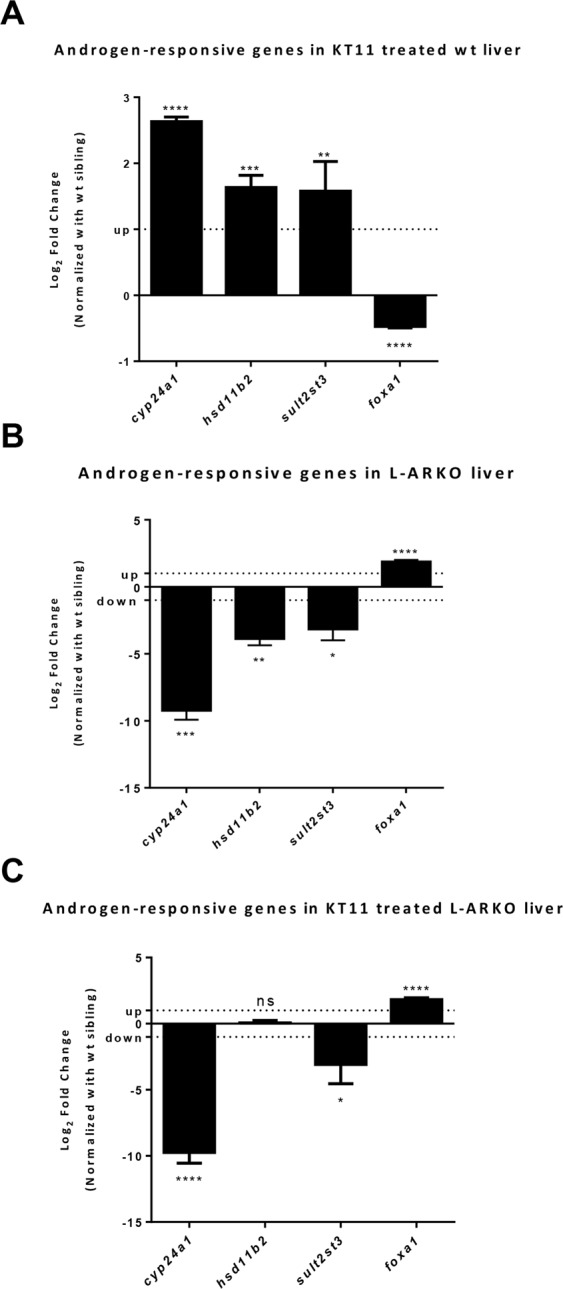
Expression of androgen-responsive genes. (**A**) Androgen-responsive gene expression in male WT zebrafish liver after one week of KT11 treatment. (**B**) Androgen-responsive gene expression in the F2 male L-ARKO zebrafish liver. (**C**) Androgen-responsive gene expression in F2 male L-ARKO liver after one week of KT11 treatment. RNA expression of selected genes was determined by RT-qPCR. Fold changes are shown in log_2_ scale in comparison to male WT control liver and dash lines indicate +1 and −1 levels. Statistical significance: *p < 0.05; **p < 0.01; ***p < 0.001; ****p < 0.0001; ns, no significant difference.

Expression of androgen-responsive genes in F2 adult L-ARKO zebrafish livers was compared with that in WT siblings to further ascertain the effects of *ar* knockout. *cyp24a1*, *hsd11b2*, *sult2st3* and *foxa1* genes were selected as androgen-responsive genes because the expressions of these genes are regulated by androgen agonist and antagonist treatments^24–27^. Indeed KT11 treatment upregulated *cyp24a1*, *hsd11b2* and *sult2st3* and downregulated *foxa1* in the liver of male WT zebrafish (Fig. 3A). In contrast, *cyp24a1*, *hsd11b2* and *sult2st3* were significantly downregulated, and *foxa1* gene was upregulated in F2 male L-ARKO zebrafish liver (Fig. 3B). Moreover, after KT11 treatment, the inducibility of the three up-regulated genes and the suppression of the down-regulated *foxa1* were largely abolished in the liver of F2 male L-ARKO fish (Fig. 3C). These results confirmed that the androgen signal was inhibited in L-ARKO fish liver.

### Effects of liver-specific *ar* knockout on liver tumor progression in *kras* zebrafish

**Figure 4:**
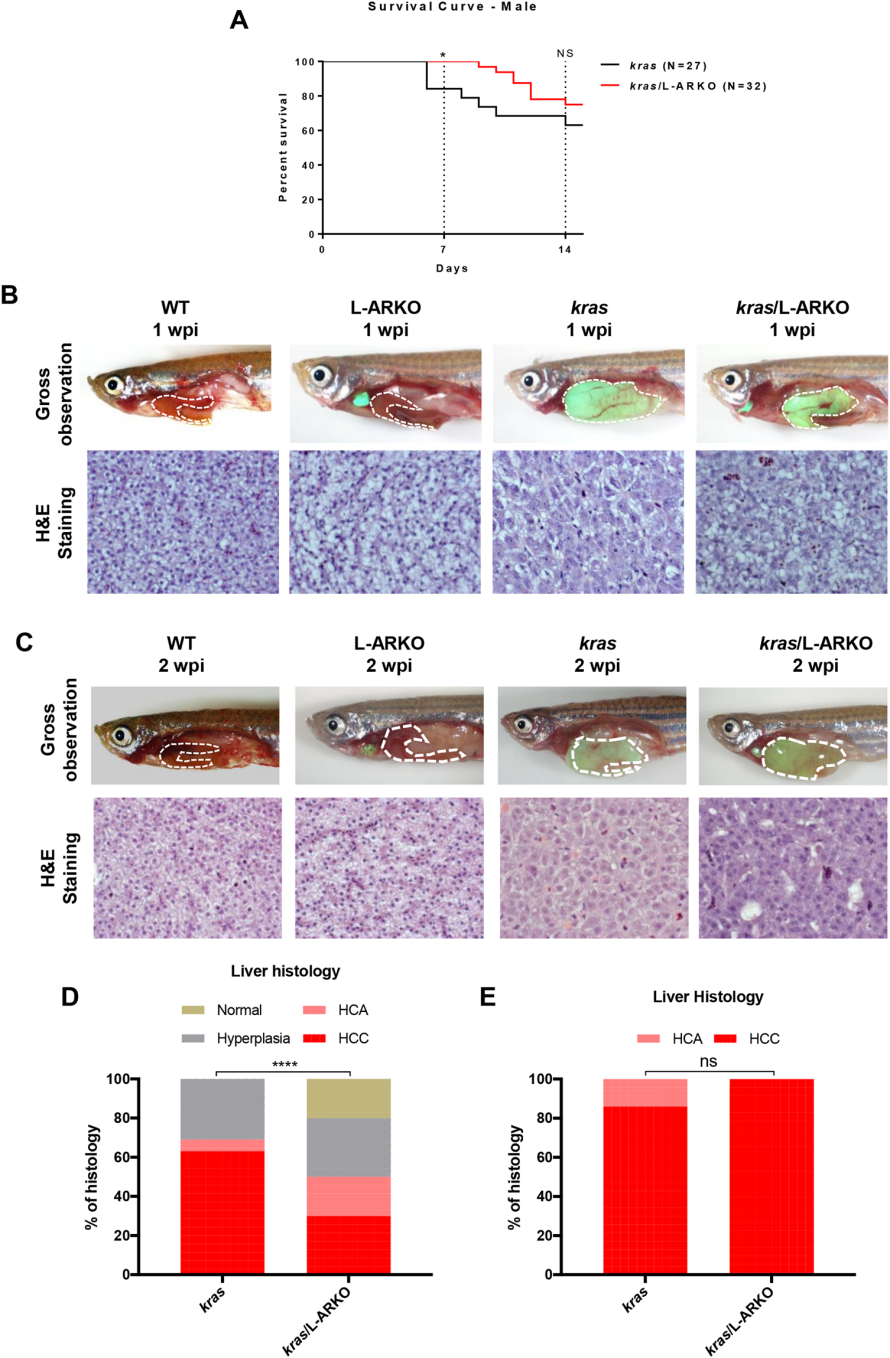
Effects of liver-specific *ar* knockout on male *kras* zebrafish. (**A**) Kaplan-Meier survival curves of male *kras* and *kras/*L-ARKO zebrafish during 14-day Dox treatment. (**B**,**C**) Representative images of the gross observations (upper) and liver histology images (lower) from WT, L-ARKO, *kras* and *kras/*L-ARKO zebrafish after Dox induction for one week (**B**) or two week (**C**). Livers are outlined by a dashed line. (**D**,**E**) Quantitative analyses of histological phenotypes of liver sections from the 1-wpi (**D**) and 2-wpi (**E**) samples. HCA, hepatocellular adenoma; HCC, hepatocellular carcinoma. Statistical significance between the *kras* and *kras*/L-ARKO groups for the incidence of different tumor types: *p < 0.05; ****p < 0.0001; ns, no significant difference.

To study the effects of liver-specific *ar* knockout in adult *kras* zebrafish on liver tumor progression, the *kras/*L-ARKO zebrafish were raised into adult. Male adult *kras*, *kras/*L-ARKO, L-ARKO and WT fish were treated with Dox for two weeks. As shown in Fig. 4A, after 1 week of Dox treatment (1 wpi, week post-induction), *kras* had significantly more deaths than that in *kras/*L-ARKO fish, but by 2 wpi, the deaths in *kras* and *kras/*L-ARKO zebrafish showed no significant difference.

At 1 wpi, there were no significant change of liver phenotype in WT and LARKO fish and normal liver histology with typical two-hepatocyte plate was observed. In contrast, majority of male *kras* zebrafish at 1 wpi showed neoplastic transformation in the liver including hyperplasia, hepatocellular adenoma (HCA) and HCC (Fig. 4B), consistent with our previous reports^28,29^. Examples of hepatic hyperplasia, HCA and HCC are shown in Supplementary Fig. S2, as classified based on established histopathological criteria^30–33^. Normal livers in zebrafish showed typical 2-cell hepatic plate structure (arrays of arrows), uniformed cell shape and size, and distinct cell boundary. Hyperplasia maintains hepatic plate arrangement but shows increased prominent nuclei (arrowheads). HCA shows unclear hepatic plates but still have clear cell boundary and relatively uniformed cell shape. HCC is characterized by the loss of cell boundaries and hepatic plate structure, as well as increase of mitotic cells and appearance of multiple nucleoli (yellow arrows).

As quantified in Fig. 4D, 62.5% of *kras* fish has HCC phenotype and the percentage was reduced to 30% in *kras*/L-ARKO fish. However, at 2 wpi, essentially all the *kras* and *kras*/L-ARKO fish had HCC phenotype while the WT and L-ARKO control fish remained normal liver morphology and histology (Fig. 4C,E). These observations suggested that the liver-specific *ar* knockout deterred liver tumor progression only at the early stage of tumorigenesis and the *ar* knockout effect was eventually overcome by the prevailing expression of oncogenic *kras*.

**Figure 5:**
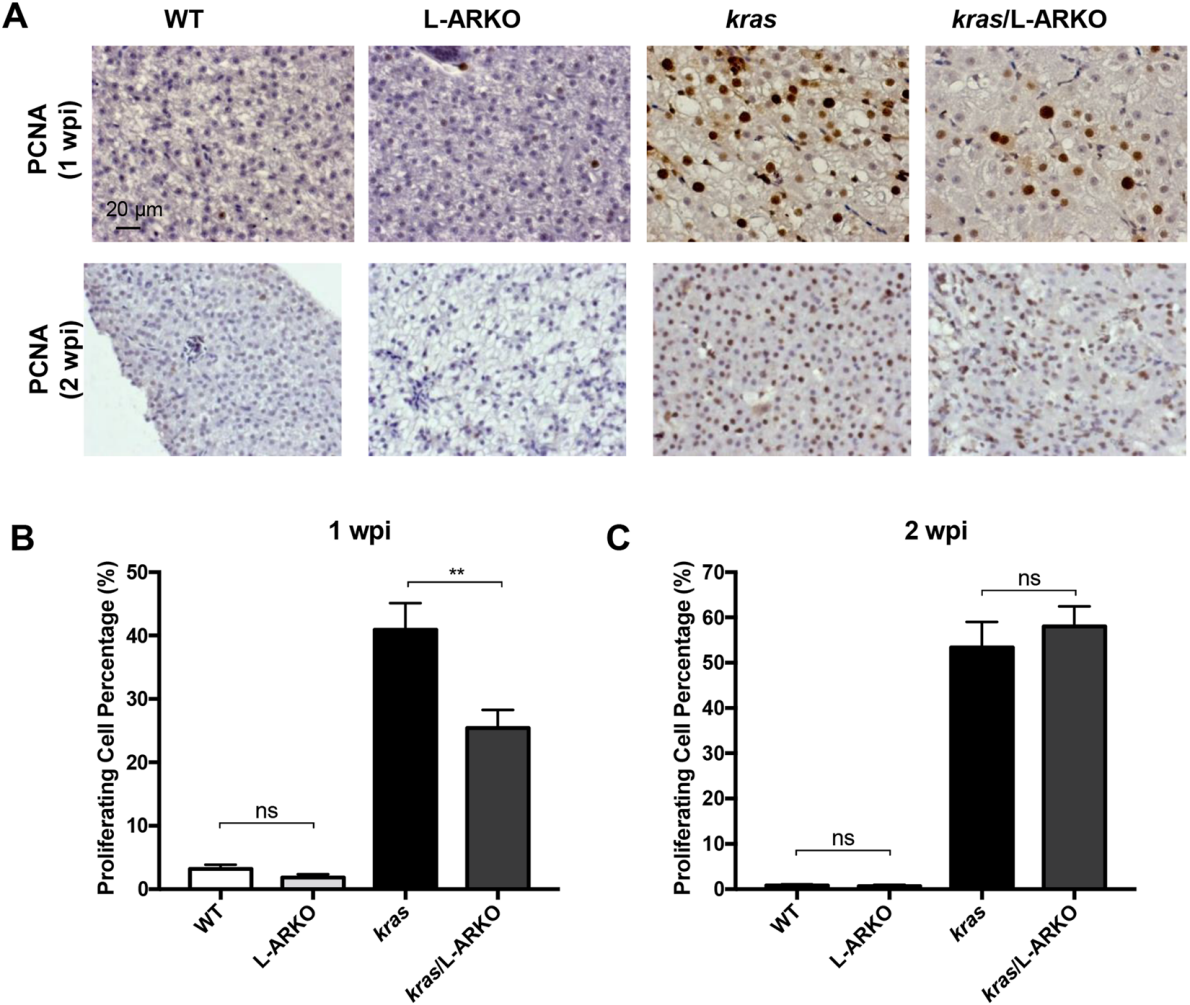
PCNA analyses of cell proliferation. (**A**) Representative images of PCNA staining of liver sections of WT, L-ARKO, *kras* and *kras/*L-ARKO zebrafish after Dox induction for one week (upper) or two week (lower). (**B**,**C**) Quantification of the cell proliferation in liver sections based on PCNA staining at 1 wpi (**B**) and 2 wpi (**C**). NS: not significant. Quantification of PCNA staining. Statistical significance: **p < 0.01; NS, not significant.

To examine cell proliferation in the liver, PCNA staining was carried out for all four groups of fish at 1 wpi and 2 wpi (Fig. 5A). In WT and L-ARKO fish, there was low background cell proliferation. However, in both *kras* and *kras*/L-ARKO fish, there were significant increases of cell proliferation. Interestingly, consistent with the histological observation, at 1 wpi, compared to *kras* fish, there was a reduced cell proliferation in L-ARKO fish (Fig. 5B), but the reduction of cell proliferation was not observed at 2 wpi (Fig. 5C). Thus, these observation further confirmed that the alleviative effect of liver-specific knockout of *ar* gene occurred only at the early stage of liver tumorigenesis.

Previously in a mouse *Ar* knockout study, it has been suggested that Ar promotes HCC via suppression of Tp53-mediated DNA damage and apoptosis, as well as increased cellular oxidative stress and DNA damage. We also examined cell apoptosis and the levels of Tp53 in the liver tumors from *kras* and *kras*/L-ARKO fish, Indeed there was an increase of cell apoptosis and in *kras*/L-ARKO fish compared to *kras* fish (Supplementary Fig. S3). These observations suggested that liver-specific knockout of *ar* attenuated liver tumor development in *kras* transgenic zebrafish involving Tp53 and cell apoptosis, in addition to cell proliferation. However, we did not find significant difference in liver tumors from *kras* and *kras*/L-ARKO fish based on staining of 8-hydroxy-2-deoxyguanosine (for cellular oxidative stress) and 4-hydroxy-2-noneal (for lipid peroxidation), two well established markers for oxidative stress^34^ (data not shown).

### Ineffectiveness of KT11 treatment on liver tumor progression in L-ARKO zebrafish

**Figure 6:**
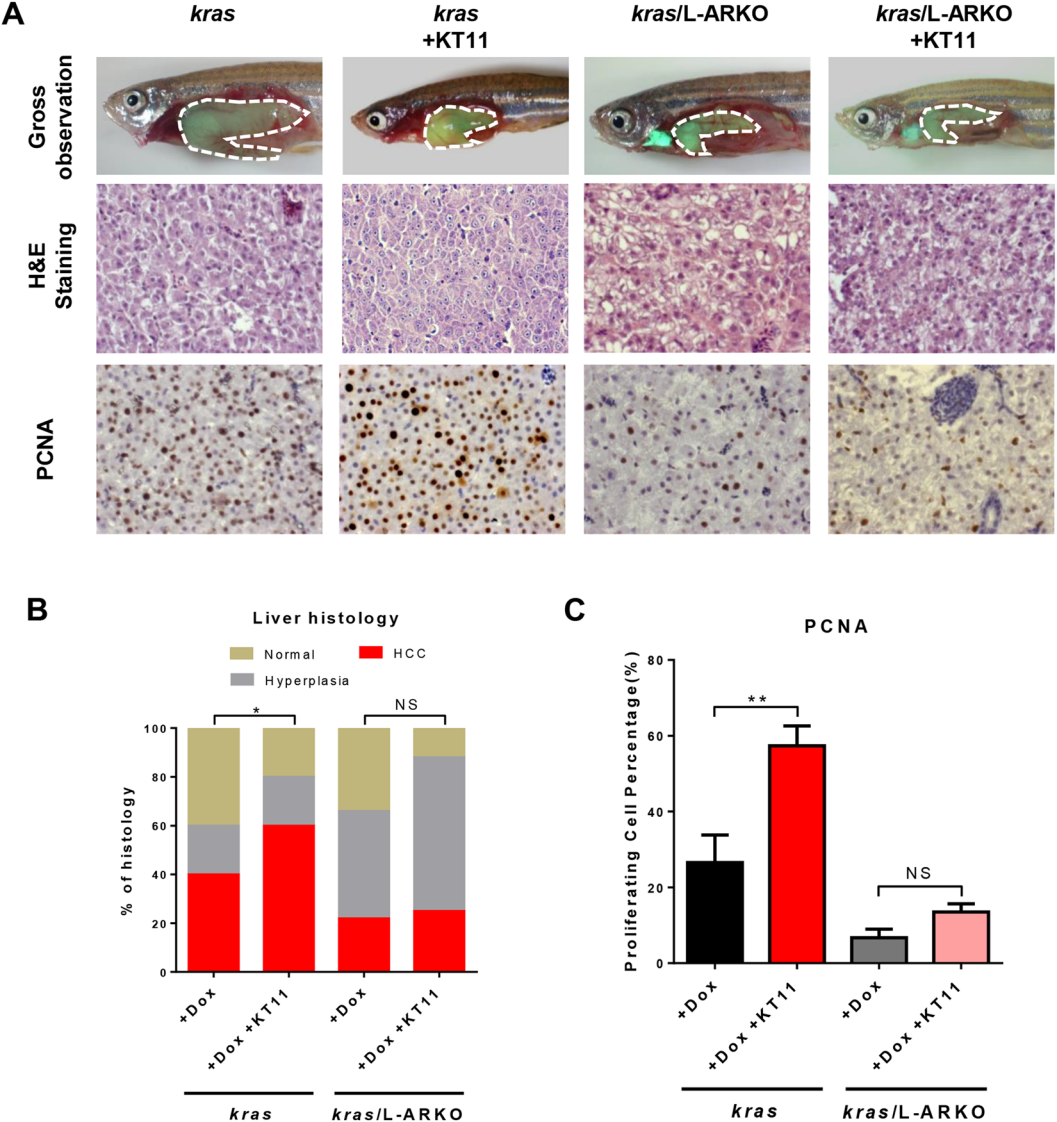
Lack of response to KT11 in *kras/*L-ARKO zebrafish during liver tumor progression. (**A**) Representative images of the gross observations (top), histology images (middle) and PCNA staining (bottom) from male *kras* and *kras/*L-ARKO zebrafish treated with Dox with or without KT11. Livers are outlined by a dashed line. (**B**) Quantitative analyses of histological phenotypes. (**C**) Quantification of liver cell proliferation based on PCNA staining. Statistical significance between the *kras* and *kras*/L-ARKO groups for the incidence of different tumor types: *p < 0.05; **p < 0.01; NS: not significant.

To further investigate the effects of liver-specific *ar* knockout on liver tumor progression, KT11 was used to treat *kras* and *kras/*L-ARKO zebrafish. At 1 wpi, HCC incidence increased after KT11 treatment in the *kras* zebrafish compared with the *kras* zebrafish with Dox alone. About 60% of the *kras* zebrafish developed HCC after KT11 treatment, while the ratio was 40% in the *kras* zebrafish with Dox alone (Fig. 6A,B). However, KT11 treatment did not increase the incidence of HCC in the *kras/*L-ARKO zebrafish in comparison with the *kras/*L-ARKO zebrafish treated with Dox alone, and the incidence of HCC in adult *kras/*L-ARKO zebrafish was lower than that in adult *kras* fish with or without KT11 treatment (Fig. 6A,B). These observations indicated that the KT11 treatment accelerated the liver tumor progression in the *kras* zebrafish, but had no effects on *kras/*L-ARKO zebrafish, suggesting the liver-specific *ar* knockout blocked the effects of KT11 during liver tumor progression.

In addition, cell proliferation in *kras* zebrafish increased significantly after KT11 treatment compared with that in the *kras* fish following Dox treatment (Fig. 6A,C), suggesting that KT11 promoted the cell proliferation during liver tumor progression. In *kras/*L-ARKO zebrafish, cell proliferation was lower than those in *kras* fish with or without KT11 treatment (Fig. 6A,C) and, after KT11 treatment, cell proliferation was not significantly increased (Fig. 6A,C). These observations suggested that the effect of KT11 was attenuated by liver-specific *ar* knockout in male *kras* zebrafish, thus further confirming the effect of *ar* knockout.

## Discussion

In the present study, the CRISPR/Cas9 system was used to generate the liver-specific *ar* knockout transgenic zebrafish using a liver (hepatocyte)-specific *fabp10a* promoter to drive *cas9* expression. *In situ* hybridization confirmed the liver-specific expression of *cas9* (Fig. 2B). Based on genomic characterization of F2 L-ARKO fish, 36% of *ar* alleles had frameshift mutations based on DNA sequencing of the whole liver DNA. Since the *fabp10a* promoter is only active in hepatocytes, the primary liver cells constituting about 70% of total number of liver cells^35^. Thus, the successful gene knockout in hepatocytes should be around 51% of total alleles. The mutagenesis rate was favourably comparable with the previous study using CRISPR/Cas9 system on zebrafish *urod* gene, in which 43% of mutated alleles was reported in FACS-sorted erythrocytes^36^. Thus, the liver-specific *ar* knockout transgenic zebrafish was successfully established using the tissue-specific CRISPR/Cas9 system and it can be used in future studies of the androgen signalling in the liver in zebrafish.

AR has been reported to play an important role in HCC progression in human and AR expression increases in the tumor tissue of HCC patients^37^. This has also been confirmed in rodents where the expression of AR is greatly increased in the liver during chemical-induced liver carcinogenesis^38^. In previous studies, AR has been demonstrated to promote hepatocarcinogenesis in HCC cell lines and some rodent models^11,14^. Previously, we also found that androgen could significantly accelerate liver tumor progression in the *kras* transgenic zebrafish model^39^. All of these works indicate the important role of androgen signalling and AR in heaptocarcinogenesis in both human and animal models.

In this study, to further investigate the contribution of Ar in liver tumorigenesis and provide a valuable experimental tool, we generated a liver-specific *ar* knockout transgenic zebrafish line, L-ARKO. by crossing L-ARKO fish with *kras* fish, we found that tumor progression in the *kras/*L-ARKO zebrafish was attenuated at the early stage (1 wpi). However, the effect of liver-specific *ar* knockout was overwhelmed by sustaining expression of the *kras* driver oncogene by 2 wpi, where essentially all *kras* fish developed HCC phenotype irrespective of the absence of *ar* gene; thus, Ar may play an important role in liver tumorigenesis only in the early stage. Consistently, cell proliferation was decreased in L-ARKO zebrafish during HCC progression at 1 wpi but not at 2 wpi, indicating that the decrease of cell proliferation might contribute to the inhibitory effect of liver-specific *ar* knockout.

Previously, a liver-specific *Ar* knockout mouse has also been established by a Cre-loxP mediated approach^14^. Similarly, hepatic knockout of *Ar* in mice caused later and less HCC. By further mechanistic studies, it was found that the Ar knockout mice had increased cellular apoptosis and Tp53, consistent with our findings in this study. Furthermore, there was also a decrease of oxidative stress in *Ar* knockout mice, but this was not observed in our zebrafish study.

Previously we have shown that liver tumorigenesis could be accelerated by androgen treatment in *kras* zebrafish^39^. In the present study, androgen treatment in *kras/*L-ARKO zebrafish showed no significant effects on liver tumor progression; thus the loss of hepatic *ar* has largely blocked the effects of androgen on HCC progression. Moreover, liver cell proliferation in the *kras/*L-ARKO zebrafish after the co-treatment of androgen and Dox showed no significant change, in contrast to the dramatic stimulation of liver cell proliferation in the androgen/Dox co-treated *kras* zebrafish, further demonstrating that the liver-specific *ar* knockout blocked the effects of androgen treatment on cell proliferation during HCC progression.

In summary, by generation of hepatocyte-specific *ar* knockout transgenic zebrafish, we have clearly shown that *ar* contributes to liver tumor progression. The loss of hepatic *ar* attenuated liver tumor progression only at the early stage through reduction of cell proliferation. However, the attenuant effect was overwhelmed by the long-term overexpression of *kras* oncogene. To determine whether the attenuation of liver tumors at the early stage by *ar* knockout is only specific to *kras*-induced liver tumors, it will be interesting to investigate if ARKO could also suppress liver tumors induced by other oncogenes. Nevertheless, our current findings are consistent with current literature in clinical studies and other experimental models that AR plays a marked role in early stages of hepatocarcinogenesis^40,41^. Furthermore, in a similar *Ar* knockout mouse model, it was also observed that there was a delayed liver tumorigenesis induced by diethylnitrosamine, a chemical carcinogen^14^. These studies together imply that the attenuation of early liver tumor by the lack of AR is likely a broad, if not universal, phenomenon.

## Methods

### Zebrafish husbandry and chemical treatments

All experiments were carried out in accordance with the recommendations in the Guide for the Care and Use of Laboratory Animals of the National Institutes of Health and the protocol was approved by the Institutional Animal Care and Use Committee (IACUC) of the National University of Singapore (Protocol Number: 096/12). Liver-specific *ar* knockout (L-ARKO) transgenic zebrafish *Tg(fabp10a::cas9; cmlc2::GFP)* were generated in the present study (see the next section). *kras*^*V12*^ transgenic zebrafish *Tg(fabp10:rtTA2s-M2; TRE2:EGFP-kras*^*G12V*^*)*, known as *kras* in the following text, were previously generated^21^. *kras*/L-ARKO double transgenic zebrafish were generated by crossing the L-ARKO and *kras* zebrafish. Treatments of adult zebrafish (4 months postfertilization) were conducted in 6-liter tanks and water with or without treatment chemicals was changed every other day. 20 mg/l doxycycline was employed to treat adult *kras*, *kras*/L-ARKO, L-ARKO and WT zebrafish siblings for 2 weeks. For hormone treatments, 5 μg/l 11-ketotestosterone (KT11) (Steraloids) was applied in conjunction with 20 mg/l doxycycline for one week.

### Design and synthesis of guide RNA for zebrafish *ar*

Guide RNA sequences were designed using a web tool CHOPCHOP (https://chopchop.rc.fas.harvard.edu/). Six *ar*-specific gRNAs were designed and one of them was selected for its high rate of germline transmission. It targeted the first exon of *ar* gene after the initiation ATG codon (Fig. 1A). The sequences of the two oligonucleotides used for the guide RNA are: forward oligo, 5′-GAAGTGCGGCTGCCTACAGGGT; Reverse oligo, 5′-CCTGTAGGCAGCCGCACTTCGA. DNA constructs were cloned through multisite gateway reaction^42^ using MultiSite Gateway™ kit (Thermo Fisher Scientific). The destination vector pDestTol2CG2-*U6:*gRNA^36^, 5′ entry vector p5E-mcs and the middle entry vector pME-Cas9 for Gateway reaction were obtained from Addgene (https://www.addgene.org). The 3′ entry vector p3E-polyA (vector #302) was requested from Tol2kit (http://tol2kit.genetics.utah.edu/index.php/P3E-polyA). A 2.8 kb promoter of the *fabp10a* gene^43^ was first cloned into pGEM T vector and then subcloned into the p5E-mcs vector. The pDestTol2CG2-*U6:gRNA* vector was digested with BseRI enzyme and annealed *ar*-specific oligos were inserted into the BseRI-digested destination vector. The multisite Gateway cloning^44^ was performed with destination vector containing gRNA, the 5′ entry vector containing the *fabp10a* promoter, the middle entry vector containing zebrafish codon-optimized Cas9 sequence and the 3′ entry vector with stop poly A signal. The resulted construct is shown in Fig. 1B. In this final construct, the *cas9* was controlled by the *fabp10a* promoter, and *ar*-specific gRNA was under the control of the ubiquitous *U6* promoter. The GFP controlled by the heart-specific *cmlc2* promoter serving as screen marker for positive transgenic fish.

### Microinjections

WT zebrafish embryos were collected for microinjection. 20 pg of DNA constructs and 20 pg of Tol2 mRNA were injected into zebrafish embryos at one-cell stage. After microinjection, embryos were raised in 0.5 × Danieau’s solution (30×: 1.74 mM NaCl, 21 mM KCl, 12 mM MgSO_4_.7H_2_O, 18 mM Ca(NO_3_)_2_, 1.5 mM HEPES buffer) at 28 °C.

### Whole-mount RNA *in situ* hybridization

*cas9* fragment was amplified using primers GAATGGATAAGGACAGTTTCA and GCTTAGCGTTTGAGAAGCT, and cloned into pGEM T easy vector (Promega). The sense and anti-sense probes were synthesized by *in vitro* transcription using mMESSAGE mMACHINE T7 Kit (Thermo Fisher Scientific). *In situ* hybridization was performed following established protocols^45^.

### T7E1 mutagenesis assay and DNA sequencing

T7E1 assay was performed following the published method^46^. Two primers were designed and synthesized based on the locations upstream and downstream of the gRNA target site: TTTTTCACGGACCTTACCAAAGC and TACTCTCGGTCTTTCCTTCCTG. For DNA sequencing, PCR products were cloned into pGEM T Easy vector (Promega) and sequenced using the ABIPRISM™ BigDye™ Terminator Cycle Sequencing Ready Reaction Kit (Perkin Elmer, USA).

### RNA extraction and real-time quantitative PCR

RNA extraction was carried out using TRIzol® Reagent (Thermo Fisher Scientific). The RNA samples were then treated with DNase I (InvitrogenTM, #18068-015) to remove possible DNA contamination and cleaned RNA was reverse-transcribed into cDNA using Transcriptor First Strand cDNA Synthesis Kit (Roche). Primers for qPCR were designed using NCBI Primer-BLAST web tool. The real-time qPCR was carried out in LightCycler® 480 Real-Time PCR System (Roche). All primers used are listed in Supplementary Table S1.

### FACS (fluorescence-activated cell sorting) isolation of hepatocytes

Zebrafish livers were freshly dissected and dissociated as previously described^47^. Through FACS by a cell sorter (BD Aria), hepatocytes were isolated based on DsRed expression from *LiPan* fish^48^. Over 10,000 cells were collected for DNA/RNA analysis. The purity of FACS isolated cells was above 90%.

### Microscopy and image analysis

The gross appearance of the liver was examined and imaged under the bright field of Olympus MVX10 stereomicroscope and photographed with an Olympus DP72 digital camera with the Olympus cellSens dimension imaging software (Olympus).

### Paraffin sectioning and histological analyses

Fish abdominal regions were dissected and fixed in formalin solution (Sigma-Aldrich), followed by dehydration and embedding into paraffin. 5 μm sections were made for hematoxylin and eosin staining. Stained slides were mounted in Micromount (Leica) and imaged using a light microscope (Zeiss, Axiovert 200 M). Classification of tumor types were based on established criteria as previously reported^32,33^. For PCNA (Proliferating cell nuclear antigen) staining, **s**ections were incubated with rabbit anti-PCNA primary antibody at 1:500 dilution, followed by incubated with horseradish peroxidase (HRP)-conjugated secondary antibodies and color development using the DAKO Real Envision Detection System.

### Statistical analyses

Statistical significance between two groups was evaluated by two-tailed unpaired Student t test (GraphPad). Statistical data were presented as mean value ± standard error of mean (SEM). P < 0.05 was chosen to be statistically significant.

## Supplementary information

Supplementary Table 1 and Supplementary Figures 1–3
