# Supplementary material for: Liver-specific androgen receptor knockout attenuates early liver tumor development in zebrafish
Source: Sci Rep. 2019 Jul 23;9:10645. doi: 10.1038/s41598-019-46378-3 (PMC6650507; doi:10.1038/s41598-019-46378-3)
Supplement: Supplementary file 1 — Supplementary Table 1 and Supplementary Figures 1–3 [file 41598_2019_46378_MOESM1_ESM.pdf]

Supplementary Information

Liver-specific androgen receptor knockout attenuates early liver tumor development in zebrafish

Hankun Li, Yan Li, Jeng-Wei Lu, Xiaojing Huo, Zhiyuan Gong

Department of Biological Sciences, National University of Singapore, Singapore

* Corresponding author, Dr. Zhiyuan Gong, Department of biological Sciences, National University of Singapore, dbsgzy@nus.edu.sg; phone: (65)2-65162860; fax: (65)-67792486.14 Science Drive 4, Singapore

Supplementary Table S1. PCR primers

Name	Sequence (5’ to 3’)
*ar*_1F	GGTCTTCAATGATCGTAGGATG
*ar*_1R	GTCTTCCTGCCATAGTTGATTA
*ar*_2F	AGTGCGGCTGCCTACAGGAG
*ar*_2R	GCTGCTTTCGGTGGCGTCTG
*cyp24a1*_F	TCCTAATGCTCAACAGTCAG
*cyp24a1*_R	TTATAGTCACGCAGAATCCA
*hsd11b2*_F	TTATCAACACACTTCGTCAC
*hsd11b2*_R	TCTCCAGCAGATATTCTTCC
*sult2st3*_F	TGGTGCCACAAGAAATTATG
*sult2st3*_R	GACTCGCTTATTATTCCTCATC
*foxa1*_F	GCAATATTCCTCCTACGGCTCA
*foxa1*_R	GAGAACAGGCCTGGAATACACA
*β-actin*_F	CCACCTTAAATGGCCTAGCA
*β-actin*_R	CATTGTGAGGAGGGCAAAGT

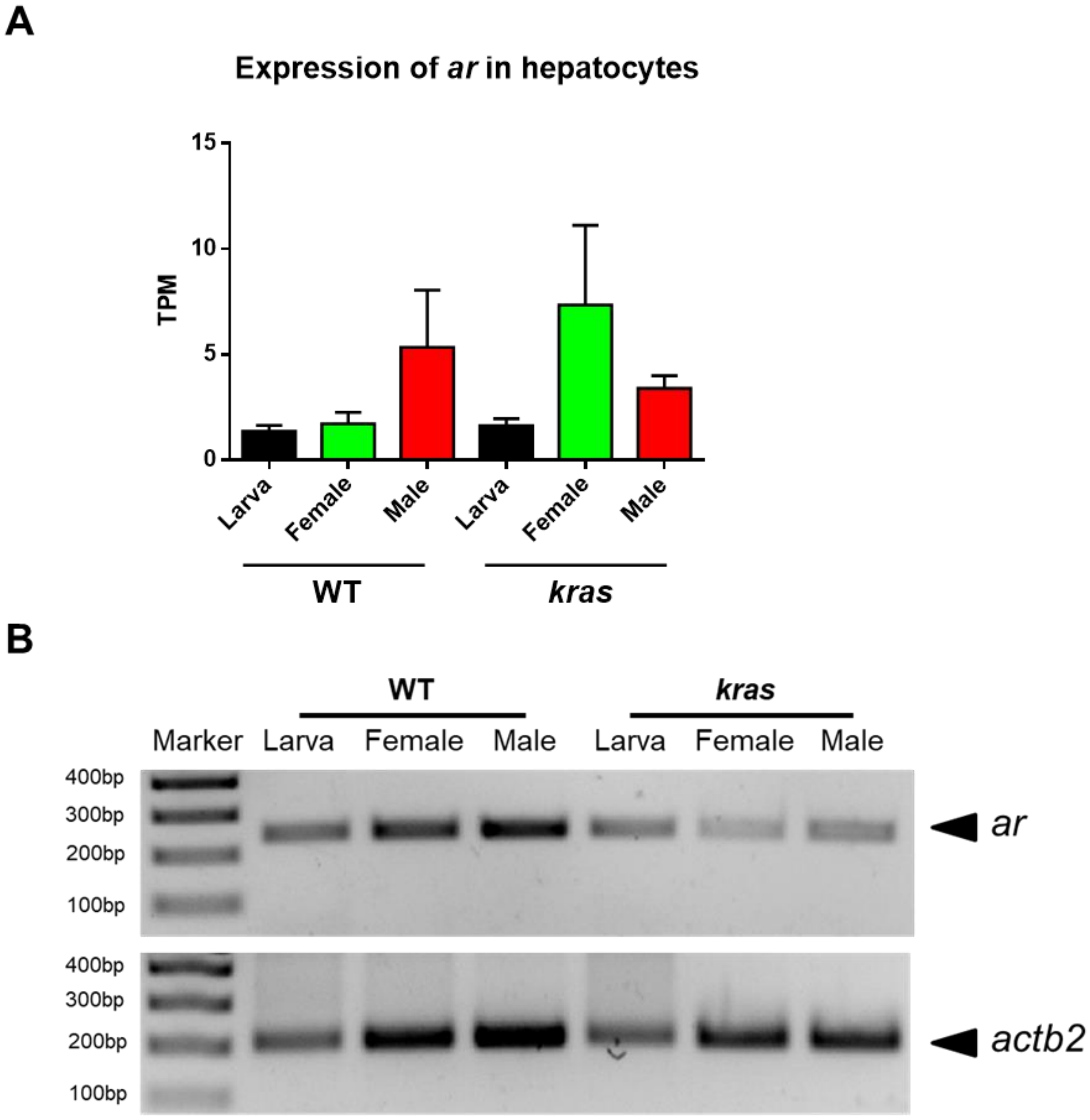

Supplementary Fig. S1. Validation of the *ar* expression in zebrafish hepatocytes in larva and adult stages. (A) Expression of *ar* based on RNA-Seq data (our unpublished) from FACS-purified hepatocytes from larvae, female and male hepatocytes of WT and *kras* zebrafish. TPM, transcripts per million. (B) RT-PCR validation of *ar* expression in the hepatocytes from the same set of larva, female and male zebrafish hepatocyte samples. *actb2* cDNA was also amplified to calibrate inputs of cDNA in RT-qPCR.

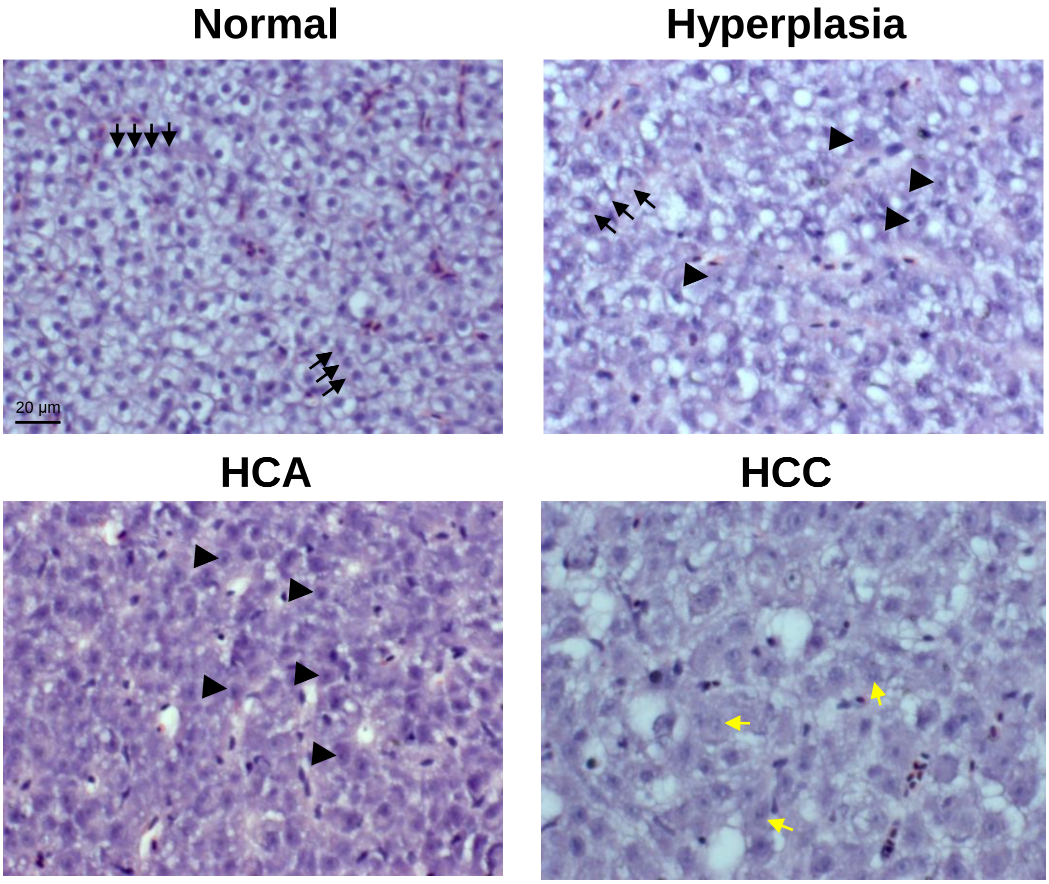

Supplementary Fig. S2. Examples of normal liver, hepatic hyperplasia, HCA and HCC. Normal livers in zebrafish showed typical 2-cell hepatic plate structure (arrays of arrows), uniformed cell shape and size, and distinct cell boundary. Hyperplasia maintains hepatic plate arrangement but shows increased prominent nuclei (arrowheads). HCA shows unclear hepatic plates but still have clear cell boundary and relatively uniformed cell shape. HCC is characterized by the loss of cell boundaries and hepatic plate structure, as well as increase of mitotic cells and appearance of multiple nucleoli (yellow arrows). Scale bar, 20 µm.

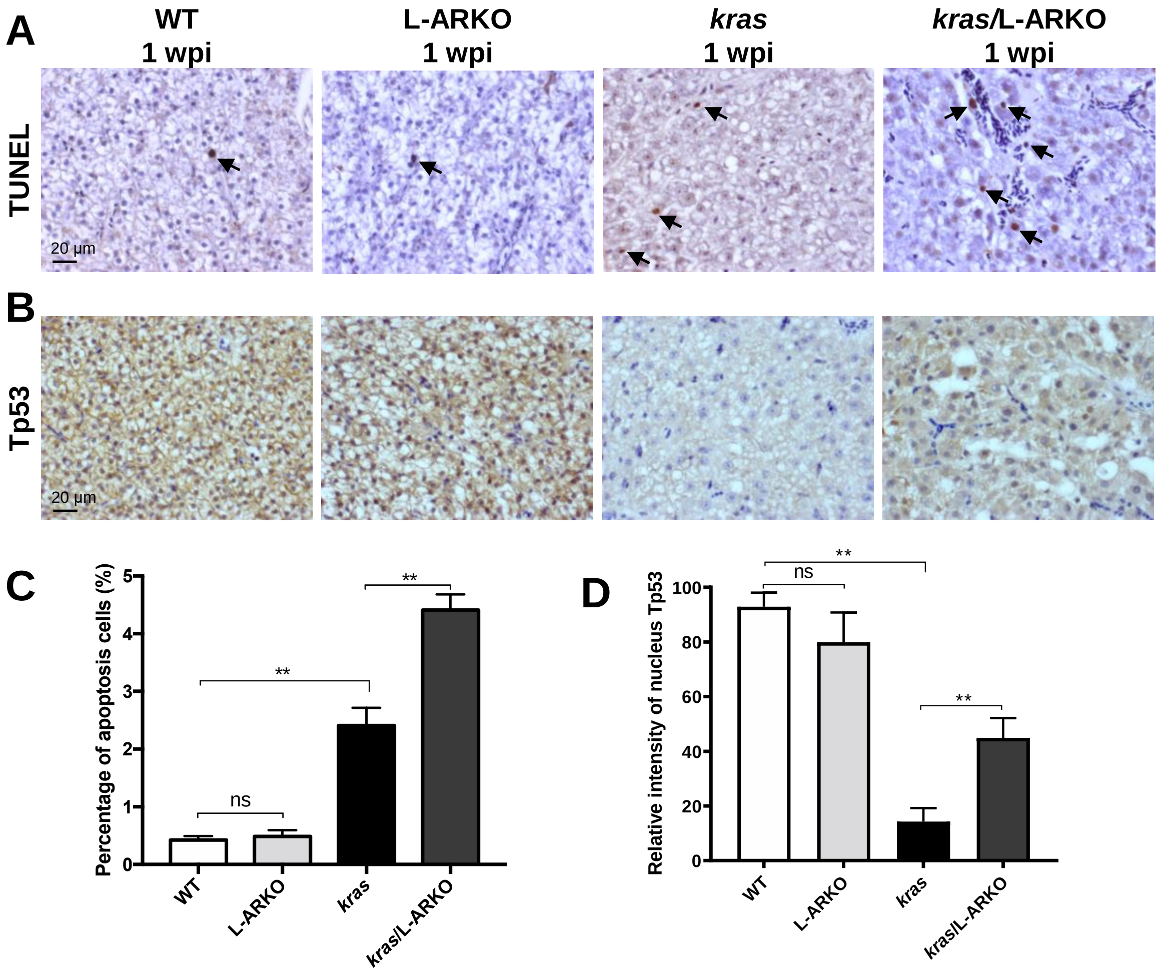

Supplementary Fig. S3. Increases of cell apoptosis and Tp53 expression in liver tumor after liver-specific knockout of *ar*. (A) TUNEL staining for cell apoptosis. Arrows point to the apoptotic cells. (B) Immunohistochemical staining for Tp53. (C) Quantification of the percentage of apoptotic cells. (D) Quantification of Tp53+ nuclei. ** $p<0.01$; ns, not significant.
